# Dynamic Effects of Early Adolescent Stress on Depressive-Like Behaviors and Expression of Cytokines and JMJD3 in the Prefrontal Cortex and Hippocampus of Rats

**DOI:** 10.3389/fpsyt.2018.00471

**Published:** 2018-10-11

**Authors:** Rui Wang, Wei Wang, Jingjing Xu, Dexiang Liu, Hong Jiang, Fang Pan

**Affiliations:** Department of Medical Psychology and Medical Ethics, Cheeloo College of Medicine, Shandong University, Jinan, China

**Keywords:** Jumonji domain-containing 3, depression, early-life stress, H3K27me3, cytokine, adolescent, epigenetic

## Abstract

**Aims:** Expression of inflammatory cytokines in the brain has been reported to be involved in the pathogenesis of and susceptibility to depression. Jumonji domain-containing 3 (Jmjd3), which is a histone H3 lysine 27 (H3K27) demethylase and can regulate microglial activation, has been regarded as a crucial element in the expression of inflammatory cytokines. Furthermore, recent studies highlighted the fact that lipopolysaccharides induce depressive-like behaviors and higher Jmjd3 expression and lower H3K27me3 expression in the brain. However, whether the process of Jmjd3 mediating inflammatory cytokines was involved in the susceptibility to depression due to early-life stress remained elusive.

**Methods:** Rats exposed to chronic unpredictable mild stress (CUMS) in adolescence were used in order to detect dynamic alterations in depressive-like behaviors and expression of cytokines, Jmjd3, and H3K27me3 in the prefrontal cortex and hippocampus. Moreover, minocycline, an inhibitor of microglial activation, was employed to observe the protective effects.

**Results:** Our results showed that CUMS during the adolescent period induced depressive-like behaviors, over-expression of cytokines, and increased Jmjd3 and decreased H3K27me3 expression in the prefrontal cortex and hippocampus of both adolescent and adult rats. However, minocycline relieved all the alterations.

**Conclusion:** The study revealed that Jmjd3 might be involved in the susceptibility to depressive-like behaviors by modulating H3K27me3 and pro-inflammatory cytokine expression in the prefrontal cortex and hippocampus of rats that had been stressed during early adolescence.

## Introduction

Depression, a major psychiatric disorder that affects approximately 16% of the entire population, has the cardinal symptoms of low mood, anhedonia, and cognitive impairment (1). A large number of studies revealed that neuroinflammation is closely related with the pathogenesis of depression (2, 3). Elevated levels of pro-inflammatory cytokines such as interleukin-1β (IL-1β) and IL-6 were observed in the periphery and cerebrum of depressed patients (4–6), and this increase could be reversed after antidepressant therapy (7). Also, some patients suffering from chronic inflammatory diseases (e.g., cardiovascular diseases) had a higher incidence rate of depression (8). In addition, 50% of the hepatitis C patients receiving interferon-α (IFN-α, a pro-inflammatory cytokine) treatment had depressive symptoms (9, 10). Our previous studies also suggested that both chronic mild stress in rats and the administration of lipopolysaccharides (LPS) in mice induced an increase in pro-inflammatory cytokines in the prefrontal cortex and hippocampus as well as depressive-like behaviors (11, 12). Those studies suggested that neuroinflammation, particularly overproduction of cytokines, plays a critical role in the etiopathogenesis of depression.

Microglial activation is one vital factor that contributes to neuroinflammation. Microglia, the primary immune cells of the brain, are regarded as the main source of inflammatory cytokines when they are activated by diverse stress (13–15). Studies have confirmed that primed microglia generally have two functional subtypes, the “classical activation” type and the “alternative activation” type (16, 17). Classical activation of microglia is closely associated with the neuroinflammatory response caused by the upregulation of pro-inflammatory cytokines [e.g., IL-1β, IL-6, tumor necrosis factor-α (TNF- α)] and free radicals such as nitric oxide, further inducing dysfunction of neural networks in the central nervous system (CNS) (18). In contrast, alternative activation of microglia releases anti-inflammatory cytokines. There exist three pathological conditions after microglial activation: (1) tissue damage, (2) inflammatory responses, and (3) hormonal disorder or stress (19). When the periphery was in an inflammatory state, pro-inflammatory cytokines (e.g., IL-1β) crossed the blood brain barrier by saturated transportation and activated microglia and later further triggered the inflammatory cascade (19). Among other factors, studies indicated that stress was a crucial contributor in the activation of microglia (13). It was reported that chronic stress induced an increase in the microglial number and caused morphological changes (20). More importantly, recent studies showed that microglia could memorize early-life stress. The memory-primed microglia had a stronger activation when reacting to stress, even to the slightest stimuli, again in later life (21). However, in the case of classical activation, the relationship between neuroinflammatory response and susceptibility to depression was unclear in the case of individuals who had been exposed to stress early in life.

Environmental stressors can modify the expression of susceptible genes, thereby, leading to the susceptibility mechanisms of depression in which histone methylation plays an important role (22). The Jumonji domain-containing 3 (Jmjd3, KDM6B), which is deemed as a histone H3 lysine 27 (H3K27) demethylase that specifically demethylates trimethylated H3K27 (H3K27me3), is associated with transcription repression. Previous studies reported that Jmjd3 could be induced by nuclear factor-kappa B (NF-κB) in response to inflammatory stimuli such as LPS (23, 24). Increased Jmjd3 could demethylate repressive H3K27me3 epigenetic marks in promoters and gene bodies. Therefore, the expression of pro-inflammatory genes was potentiated, thereby, causing an inflammatory status in the CNS (23–26). Meanwhile, GSK-J4, as a selective Jmjd3 inhibitor, could limit the inflammation accompanied by a reduction in the levels of pro-inflammatory cytokines (27, 28).

Based on all the above studies, the present study first hypothesized that early-life stress induced susceptibility to depressive-like behaviors and expression of pro-inflammatory cytokines by the activation of microglia in the brain. Second, our study hypothesized that Jmjd3 was involved in the susceptibility to those alterations by modulating pro-inflammatory cytokines. In this study, chronic unpredictable mild stress (CUMS) was used to establish an animal model of depression in adolescence. Depressive-like behaviors, pro-inflammatory cytokine expression, microglial activation, as well as Jmjd3 and H3K27me3 expression in the prefrontal cortex and hippocampus were determined by a sucrose preference test (SPT), open field test (OFT), elevated plus maze (EPM), and Morris water maze (MWM), as well as by immunohistochemistry and immunoblotting. The purpose of this study was to evaluate the dynamic effects of CUMS in early-life on behavioral changes, cytokine expression, and Jmjd3 and H3K27me3 expression. As a second generation semi-synthetic tetracycline antibiotic, minocycline plays a neuroprotective role in many neuroinflammatory diseases of the CNS (29). It has been reported that minocycline is a microglial activation inhibitor and can suppress the secretion of pro-inflammatory cytokines such as IL-1β (30) and relieve stress-induced depressive- and anxiety-like behaviors in adult rats (31). Our goal was to investigate the effects of minocycline on relieving behavioral dysfunction, cytokine expression, and Jmjd3 and H3K27me3 expression in stressed adolescent and adult rats.

## Materials and methods

### Animals

Sixty male 21-day-old Wistar rats were obtained from the Experimental Animal Center of Shandong University and were housed in groups of five, with each cage maintained under standard laboratory conditions (12 h light/dark cycle, 25°C), with food and water provided *ad libitum* during the study. All procedures of this study were carried out with the approval of the Animal Ethics Committee of Shandong University.

### Experimental design

After a 7-day acclimatization, the rats were randomly divided into three groups (*n* = 20 in each group): control group (C), CUMS group (S), CUMS and minocycline group (S+M). The C group was the normal control, whereas the S group was exposed to CUMS for 3 weeks. The S+M group received both the CUMS procedure and the minocycline treatment. After 3 weeks of drug administration and animal modeling, 10 rats from each group were randomly selected to undergo behavioral tests. The order of the behavioral tests was as follows: the SPT, then the OFT, the EPM test, and finally the MWM test. Later, at the age of 55 days, the 30 selected rats were sacrificed. The remaining 30 rats were raised to adulthood, and then they underwent the behavioral tests. Afterwards, these rats were sacrificed at the age of 90 days (see Supplementary Material). Therefore, our study had six groups altogether: the adolescent control group (AdoC); the adolescent CUMS group (AdoS); the adolescent CUMS and minocycline group (AdoS+M); the adult control group (AduC); the adult CUMS group (AduS); and the adult CUMS and minocycline group (AduS+M).

### CUMS procedure and drug administration

The CUMS was applied to all groups except for the control group, using a previously described method (32, 33). Rats were exposed to one of the following seven stressors randomly every day: food deprivation (24 h), water deprivation (24 h), radio noise in the room (8 h), heat stress (45°C, 5 min), foot shock (30 mV, 10 s duration for a total of 10 min), light/dark cycle reversal, and pinching tail (1 min) (see Table 1). Rats in the S+M group were treated with minocycline (intragastric, 40 mg/kg, diluted in saline) 30 min before CUMS for 3 weeks (17).

**Table 1:** Schedule of unpredictable chronic mild stress.

**Time**	**Type of stress**
Day 1	Heat stress (45°C, 5 min)
Day 2	Light/dark cycle reversal
Day 3	Radio noise in the room (8 h)
Day 4	Water deprivation (24 h)
Day 5	Pinching tail (1 min)
Day 6	Food deprivation (24 h)
Day 7	Foot shock (30 mV, 10 s duration for a total of 10 min)
Day 8	Radio noise in the room (8 h)
Day 9	Heat stress (45°C, 5 min)
Day 10	Food deprivation (24 h)
Day 11	Pinching tail (1 min)
Day 12	Water deprivation (24 h)
Day 13	Foot shock (30 mV, 10 s duration for a total of 10 min)
Day 14	Light/dark cycle reversal
Day 15	Food deprivation (24 h)
Day 16	Pinching tail (1 min)
Day 17	Radio noise in the room (8 h)
Day 18	Light/dark cycle reversal
Day 19	Heat stress (45°C, 5 min)
Day 20	Foot shock (30 mV, 10 s duration for a total of 10 min)
Day 21	Water deprivation (24 h)

### Behavioral tests

#### Sucrose preference test (SPT)

The SPT was used to evaluate the level of anhedonia (34). Rats were trained to adapt to the 1% sucrose solution in a quiet environment before the test. In the first 24 h, each cage was provided with two identical bottles of the sucrose solution. Subsequently, in the second 24 h, each cage was provided with one bottle containing water and another bottle with the sucrose solution. After 48 h of adaptation, the rats were housed alone and deprived of water and food for 24 h. Later, two pre-weighed bottles with water and sucrose solution, respectively, were simultaneously placed in each cage. After 1 h, the two bottles were weighed again and the consumption was calculated. The sucrose preference was expressed as the sucrose preference (%), which was calculated as [sucrose consumption/(sucrose consumption + water consumption)].

#### Open field test (OFT)

The OFT was used to determine the autonomous and exploratory behaviors of rats in a novel environment (35). The apparatus for the OFT was a white wooden box without a top structure (100 cm diameter and 50 cm wall height). The bottom of the apparatus was divided into 25 squares. The rats were placed one at a time into the central square and were allowed to freely explore the field for 5 min. The locomotion activity (the number of crossings), rearing, and grooming behaviors of the rats were recorded by a SMART video tracking system (SMART v3.0, Panlab, Spain). After each test, we cleaned the device with alcohol before inserting the next rat.

#### Elevated plus maze (EPM)

The EPM was used to test the anxiety level of the rats (36). The apparatus was elevated 50 cm from the ground. Two opposing open arms (30 cm long × 15 cm high) and two closed arms formed a cross around the central platform (5 cm × 5 cm). Each rat was individually placed onto the central platform facing an open arm and was allowed to freely explore the maze for 5 min. The number of entries into each arm was reported by the video tracking system (SMART v3.0, Panlab, Spain). A lower ratio of open arm entries to total entries signified a higher level of anxiety in the rat. We cleaned the apparatus after each test before inserting the next rat.

#### Morris water maze (MWM)

The MWM was used to determine the spatial memory and the learning ability of rats (37). The apparatus was a circular black tank (120 cm diameter and 50 cm height) and was filled with warm water (22°C). The software assigned four quadrants to the surface of the water. The platform (13 cm diameter and 29 cm height) was placed at the center of the target quadrant (quadrant II) and was 1–2 cm below the surface of the water. The movement of each rat was tracked and recorded using the SMART video tracking system (SMART v3.0, Panlab, Spain). A training period was carried out for 5 consecutive days, four times a day. The rats were gently placed into a random quadrant facing the wall and were allowed to swim freely to find the platform within 60 s, followed by a 20-s reprieve on the platform. Rats that failed to find the platform were manually placed on the platform for a 20-s break. The sixth day was the testing period; the rats were placed into quadrant IV and allowed to swim freely for 60 s in the maze without the platform. The number of across the target quadrant and the platform were recorded.

### Pro-inflammatory cytokine expression analysis

#### Sample collection

Five rats from each group were randomly selected after completing the behavioral tests and then were decapitated immediately after being anesthetized. The whole brain was taken out of the skull carefully after opening the skull along the sagittal suture. Both sides of the prefrontal cortex and the hippocampus were isolated, and the samples were stored at −80°C. All the processes were performed on ice.

#### Protein extraction

Lysis buffer and 1% protease inhibitor (phenylmethanesulfonyl fluoride) were added according to the weight of the sample. The tissue homogenate was centrifuged at 12,000 rpm for 25 min at 4°C, and then, the supernatant was extracted. The protein concentrations were determined by a Micro Bicinchoninic Acid Protein Assay Kit (Beyotime Institute of Biotechnology, China).

#### Enzyme-linked immunosorbent assay (ELISA)

According to the ELISA kit instruction book (Tianjin Anoric Bio-technology Co., Ltd, China), different concentrations of standards and samples were added in a particular order to the 96-coated well plates. Subsequently, a biotin-antibody diluent was added to the samples, followed by 100 μL of an enzyme conjugation liquid. After incubation at 37°C for 60 min, the coated microwell plates were washed five times with a prepared washing liquid. The solutions of TMB I, TMB II, and stopping liquid were added to the plates in turn. The iMark Microplate Absorbance Reader (Bio-Rad Labs, Hercules, CA, USA) was used to detect the optical density at 450 nm. According to the standard curve drawn from the optical density of the standard, the concentration of each sample was calculated.

### Histological measurements

In order to observe the changes in microglial number and morphology, ionized calcium-binding adapter molecule 1 (Iba-1), a microglial marker, was detected by immunofluorescence as in a previous study (31). Five rats in each group were randomly chosen to receive a heart perfusion with 50 mL saline and 100 mL 4% paraformaldehyde (PFA) in phosphate buffer (0.1 M, pH 7.4) after being deeply anesthetized with pentobarbital. After that, the brains were removed and fixed in 4% PFA for 24 h at 4°C. After paraffin embedding, the brain samples were cut into 4 μm sections. The sections were incubated in citrate buffer (pH 6.0) to facilitate antigen retrieval at high temperature. The tissues were uniformly covered with 3% bovine serum albumin (BSA) and were blocked for 30 min at room temperature. Afterwards, the sections were incubated with rabbit anti-Iba1 antibody (1:200, Abcam, USA) at 4°C overnight. After washing thrice with phosphate-buffered saline (PBS), the sections were incubated with a secondary antibody (goat anti-rabbit, Alexa 594 conjugated, 1:1000, Invitrogen, USA) for 50 min in the dark. After three washes in PBS, the nuclei were stained with 4′,6-diamidino-2-phenylindole (DAPI) for 10 min in the dark. Later, the sections were sealed with an anti-fluorescence quenching sealant and observed under a Nikon Eclipse TI-SR microscope. The images were captured with a color camera (Nikon DS-U3).

The expression of inducible nitric oxide synthase (iNOS) was determined using immunohistochemical analysis. The paraffin sections were placed in citrate buffer (pH 6.0) in a microwave oven for antigen retrieval. After natural cooling, the sections were removed and washed with water and PBS. Subsequently, the sections were incubated with 3% H_2_O_2_ for 10 min and were blocked with 3% BSA for 30 min. Next, the sections were incubated with rabbit anti-iNOS antibody (1:200, Abcam, USA) at 4°C overnight. After washing thrice with PBS, the sections were incubated with a biotinylated secondary antibody at 37°C for 30 min. After washing with PBS, the sections were incubated with a streptavidin-biotin complex. Later, the sections were dyed with diaminobenzene and observed microscopically. The images were captured with the above mentioned microscope camera.

### Epigenetic markers and iNOS in the western blot analysis

Sample collection and protein extraction were performed in a manner similar to ELISA. Protein concentrations were mixed with a 5 × Laemmli loading buffer and then were heated at 100°C for 5 min. Subsequently, 20 μg of the protein sample was loaded in a prepared sodium dodecyl sulfate-polyacrylamide gel (5% stacking gel and 8 or 10% resolving gel, according to the molecular weight of the protein) and was separated by electrophoresis. The protein was electro-transferred to polyvinylidene difluoride (PVDF) membranes (Bio-Rad, CA, USA). The PVDF membranes were blocked with 5% defatted milk in a Tris buffered saline with 0.1% Tween-20 (TBST) for 90 min at room temperature and were incubated with primary antibodies against iNOS (1:500, Abcam, USA), Jmjd3 (1:800, Abcam, USA), H3K27me3 (1:1000, Biogot Technology, Co., Ltd), and β-actin (1:5000, Biogot Technology, Co., Ltd) at 4°C overnight. The secondary antibodies (1:10000, ZSGB-BIO, China) were incubated for 60 min at room temperature. After washing thrice with TBST, the PVDF membranes were incubated with a prepared enhanced chemiluminescence mixture (Millipore Corp, Billerica, MA, USA) for 1 min and were visualized on film in the dark. The gray value of the bands was quantified with the Image J 14.0 software.

### Statistical analysis

All experimental data were presented as the mean ± SEM. The SPSS 17.0 software was utilized to analyze the data. The adolescent groups and adult groups were analyzed separately. Data were analyzed using a one-way analysis of variance followed by Tukey's *post hoc* test. Significance levels were set at *p* < 0.05.

## Results

### Behavioral test

#### Comparison of depressive-like behavior and anxiety-like behavior alterations between groups

**Figure 1:**
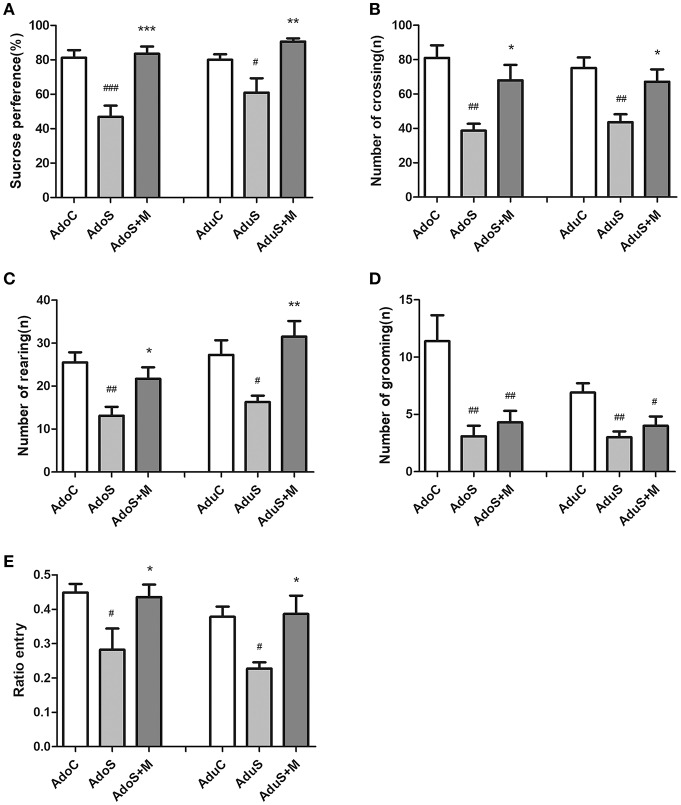
CUMS induced depressive-like behaviors, while minocycline treatment reversed the alteration in both adolescent and adult rats. **(A)** Sucrose preference percentage in the SPT; **(B)** Number of crossing in the OFT; **(C)** Number of rearing in the OFT; **(D)** Number of grooming in the OFT; **(E)** Open arm entries ratio in the EPM test; Results were expressed as the mean ± SEM (*n* = 9 or 10 in each group). ^#^, ^##^, and ^###^ indicate *p* < 0.05, *p* < 0.01, and *p* < 0.001 vs. C group, respectively; ^*^, ^**^, and ^***^ indicate *p* < 0.05, *p* < 0.01, and *p* < 0.001 vs. S group, respectively. AdoC, adolescent control group; AdoS, adolescent CUMS group; AdoS+M, adolescent CUMS and minocycline group; AduC, adult control group; AduS, adult CUMS group; AduS+M, adult CUMS and minocycline group.

Figure 1A illustrates the results of the SPT. In the adolescent groups, the percentage of sucrose consumption [*F*_(2, 27)_ = 16.08, *p* < 0.001] in the AdoS group was lower than the AdoC group (*p* < 0.001) and the AdoS+M group (*p* < 0.001). The same results could be seen in the adult groups; the percentage of sucrose consumption in the AduS group [*F*_(2, 27)_ = 8.21, *p* = 0.002] was lesser than the AduC group (*p* = 0.041) and the AduS+M group (*p* = 0.001). The results indicated that stress in early adolescence decreased sucrose preference in both adolescent and adult rats, but treatment with minocycline could reverse the decrease in sucrose consumption.

Figures 1B–D illustrate the results of the OFT. Figure 1B shows the number of crossings in the OFT for the adolescent groups [*F*_(2, 27)_ = 9.47, *p* = 0.001] and adult groups [*F*_(2, 27)_ = 7.40, *p* = 0.003], respectively. The number of crossing in the AdoS group was lesser than the AdoC group (*p* = 0.001) and the AdoS+M group (*p* = 0.017). Similarly, the AduS group had a lesser number of crossing than the AduC group (*p* = 0.003). Treatment with minocycline resulted in an increased number of crossing in the AduS+M group (*p* = 0.027) when compared with the AduS group. Figure 1C shows the number of rearings in the OFT for the adolescent groups [*F*_(2, 27)_ = 7.05, *p* = 0.003] and adult groups [*F*_(2, 27)_ = 6.91, *p* = 0.004]. In the adolescent groups, the AdoS group showed lower numbers of rearing than the AdoC group (*p* = 0.003). Moreover, treatment with minocycline normalized the behavioral deficit when compared with the AdoS group (*p* = 0.044). In the adult groups, the number of rearing in the AduS group was lower than the AduC group (*p* = 0.038) and the AduS+M group (*p* = 0.004). Figure 1D shows the amount of grooming in the adolescent groups [*F*_(2, 27)_ = 8.72, *p* = 0.001] and adult groups [*F*_(2, 27)_ = 7.65, *p* = 0.002]. Both the AdoS group and the AduS group had lesser number of grooming than the AdoC group (*p* = 0.002) and the AduC group (*p* = 0.002), respectively. However, minocycline treatment had no effect on the amount of grooming either in adolescent or in adult rats. These results indicate that stress reduced automatic and exploratory behaviors in both adolescent and adult rats. Moreover, as an inhibitor of microglial activation, minocycline normalized most of the behavioral changes in both adolescent and adult rats.

Figure 1E shows the ratio of entry into the open arm in the EPM test in the adolescent and adult groups. The AdoS group [*F*_(2, 26)_ = 4.63, *p* = 0.019] had a significantly lower ratio of entry than the AdoC group (*p* = 0.027) and the AdoS+M group (*p* = 0.045). Similarly, there was a significant reduction of open arm entries ratio in the AduS group [*F*_(2, 27)_ = 5.87, *p* = 0.008] when compared with the AduC group (*p* = 0.02) and the AduS+M group (*p* = 0.014). These results indicate that stress in early adolescence induces anxiety-like behaviors in both adolescent rats and adult rats and that minocycline treatment improves abnormal behaviors.

#### Comparison of cognitive impairment in the MWM test between groups

**Figure 2:**
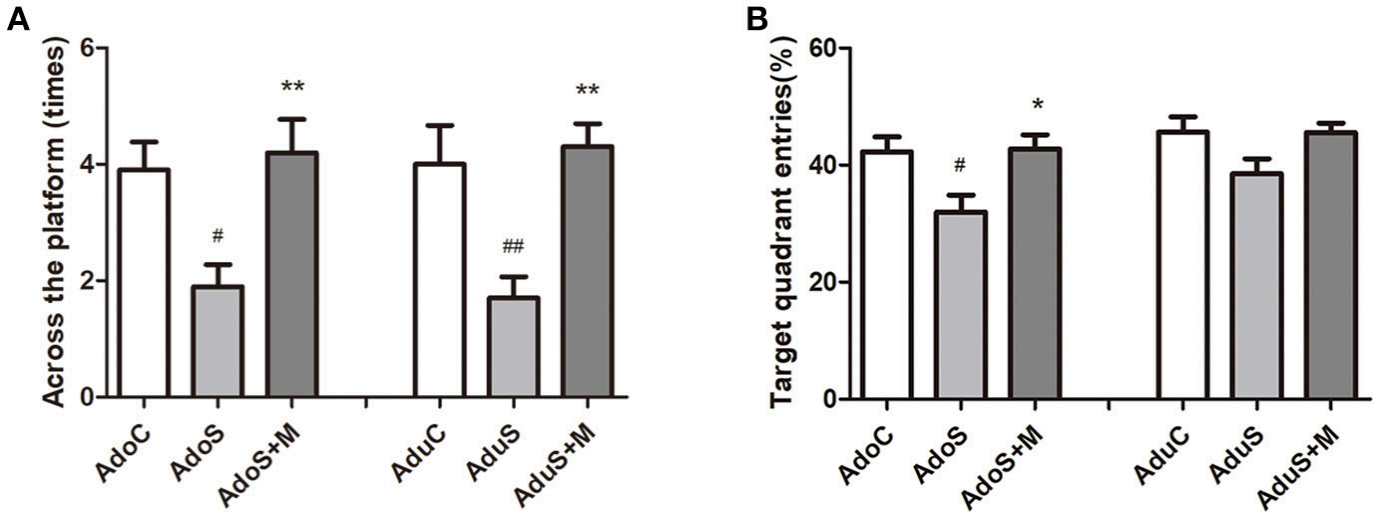
CUMS induced spatial learning and memory impairment, while minocycline treatment reversed the alteration in both adolescent and adult rats. **(A)** Number of crossings of the platform in the MWM test; **(B)** Target quadrant entries percentage in the MWM test. Results are expressed as the mean ± SEM (*n* = 10 in each group). ^#^ and ^##^ indicate *p* < 0.05 and *p* < 0.01 vs. C group, respectively; ^*^ and ^**^ indicate *p* < 0.05 and *p* < 0.01 vs. S group, respectively. AdoC, adolescent control group; AdoS, adolescent CUMS group; AdoS+M, adolescent CUMS and minocycline group; AduC, adult control group; AduS, adult CUMS group; AduS+M, adult CUMS and minocycline group.

Figure 2 presents the results of the MWM test. Figure 2A presents the number of times through the platform during the probe test in the adolescent groups [*F*_(2, 27)_ = 6.66, *p* = 0.004] and adult groups [*F*_(2, 27)_ = 8.25, *p* = 0.002]. Compared with the AdoC group (*p* = 0.019) and the AdoS+M group (*p* = 0.006), the number of times through the platform decreased significantly in the AdoS group. The AduS group also had lower number of times through the platform than the AduC group (*p* = 0.008) and the AduS+M group (*p* = 0.003). Figure 2B shows the percentage of entries into the target quadrant in the adolescent groups [*F*_(2, 27)_ = 5.47, *p* = 0.01] and adult groups [*F*_(2, 27)_ = 3.11, *p* = 0.061]. The AdoS group had reduced percentage of entries when compared with the AdoC group (*p* = 0.024) and the AdoS+M group (*p* = 0.018). However, there were no significant differences in the three adult groups. These results suggest that stress in early adolescence induced learning and memory impairment, and the inhibitor of microglial activation could reverse the damages.

### Comparison of pro-inflammatory cytokine expression in the prefrontal cortex and hippocampus between groups

**Figure 3:**
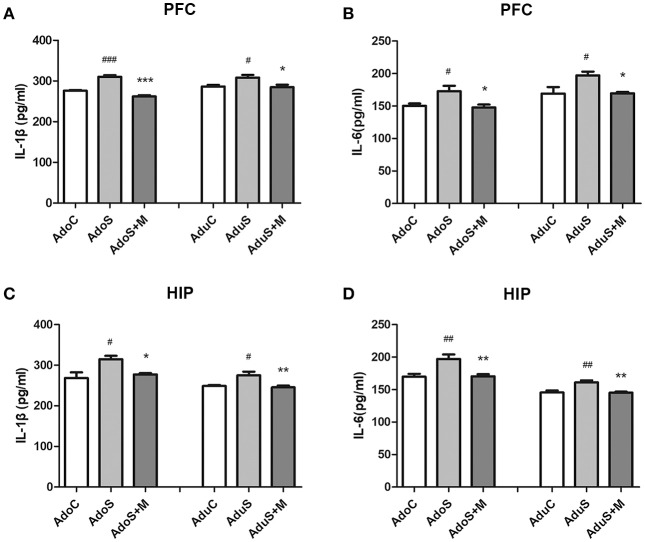
The levels of cytokines in the prefrontal cortex and hippocampus of both the adolescent and adult groups. **(A)** IL-1β expression in the prefrontal cortex; **(B)** IL-6 expression in the prefrontal cortex; **(C)** IL-1β expression in the hippocampus; **(D)** IL-6 expression in the hippocampus. Results are expressed as the mean ± SEM (*n* = 5 in each group). ^#^, ^##^, and ^###^ indicate *p* < 0.05, *p* < 0.01, and *p* < 0.001 vs. C group, respectively; ^*^, ^**^, and ^***^ indicate *p* < 0.05, *p* < 0.01, and *p* < 0.001 vs. S group, respectively. AdoC, adolescent control group; AdoS, adolescent CUMS group; AdoS+M, adolescent CUMS and minocycline group; AduC, adult control group; AduS, adult CUMS group; AduS+M, adult CUMS and minocycline group.

Figures 3A,B show cytokine expression in the prefrontal cortex. As Figure 3A shows, the levels of IL-1β increased in both the AdoS group [*F*_(2, 12)_ = 60.57, *p* < 0.001] (*p* < 0.001) and the AduS group [*F*_(2, 12)_ = 5.63, *p* = 0.019] (*p* = 0.04), when compared with the AdoC group and the AduC group, respectively. However, these increases were attenuated by minocycline treatment in both adolescent and adult rats (*p* < 0.001; *p* = 0.027). Figure 3B shows the levels of IL-6 in the prefrontal cortex. The AdoS group had increased levels of IL-6 when compared with the AdoC group [*F*_(2, 12)_ = 5.60, *p* = 0.019] (*p* = 0.045) and the AdoS+M group (*p* = 0.026). The same change tendency was observed in the adult groups. There was an increased level of IL-6 in the AduS group when compared with the AduC group [*F*_(2, 12)_ = 5.58, *p* = 0.019] (*p* = 0.032) and the AduS+M group (*p* = 0.035).

Figures 3C,D show cytokine expression in the hippocampus. Figure 3C shows the levels of IL-1β in both the adolescent groups [*F*_(2, 12)_ = 6.64, *p* = 0.011] and the adult groups [*F*_(2, 12)_ = 7.74, *p* = 0.007]. The same change tendency that was observed in the prefrontal cortex was seen in the hippocampus; the AdoS group and the AduS group had higher levels of IL-1β than the AdoC group (*p* = 0.013) and the AduC group (*p* = 0.02), respectively. Minocycline decreased the levels of IL-1β in both adolescent (*p* = 0.042) and adult (*p* = 0.009) rats. Figure 3D shows that the levels of IL-6 increased in both the AdoS group [*F*_(2, 12)_ = 9.91, *p* = 0.003] (*p* = 0.005) and the AduS group [*F*_(2, 12)_ = 11.03, *p* = 0.002] (*p* = 0.004), when compared with the AdoC group and the AduC group, respectively. Minocycline decreased the levels of IL-6 in both adolescent (*p* = 0.007) and adult (*p* = 0.004) rats.

### Comparison of microglial activation in the prefrontal cortex and hippocampus between groups

**Figure 4:**
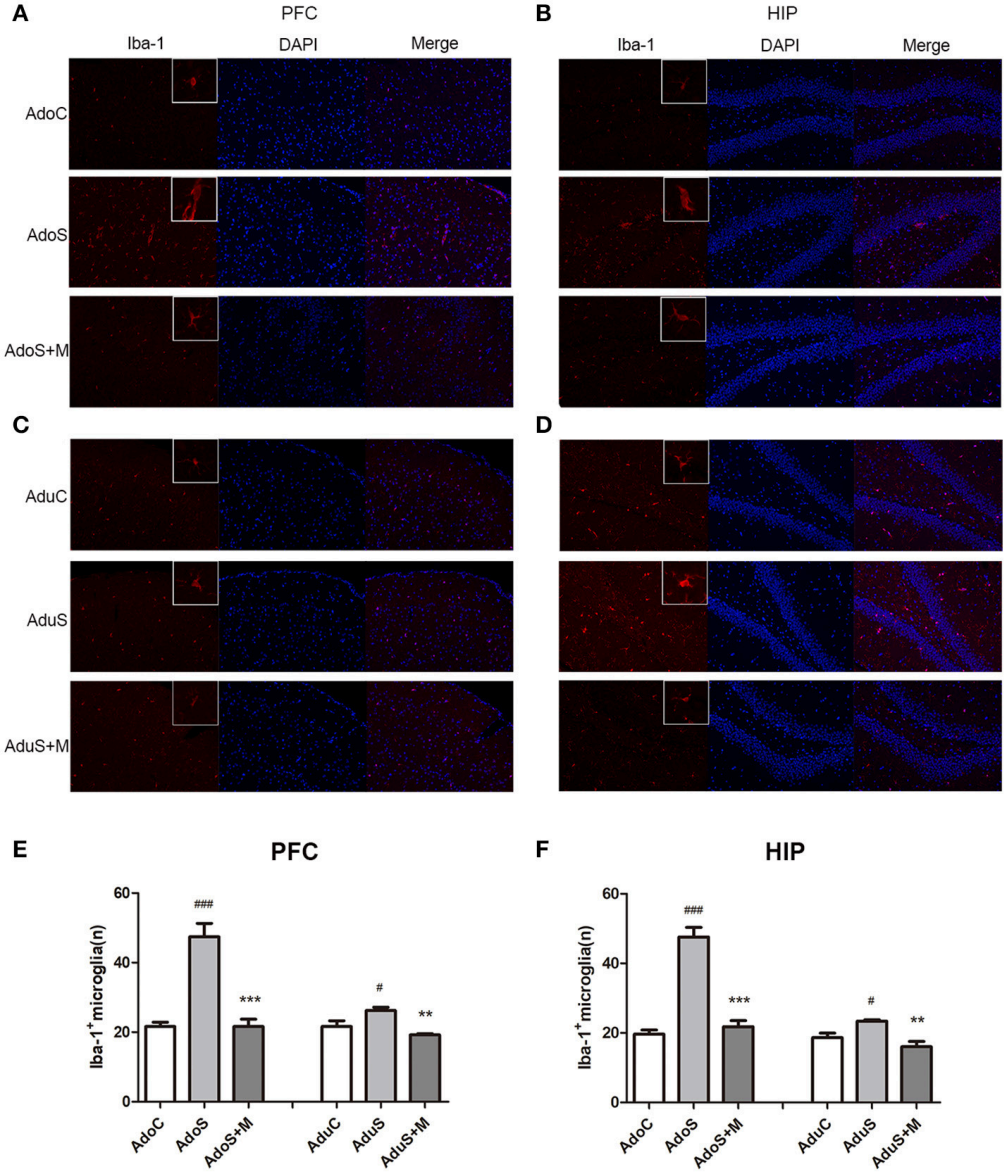
CUMS induced microglial activation in the prefrontal cortex and hippocampus of both the adolescent and adult rats, while minocycline reduced the alteration. **(A)** Iba-1/DAPI (red/blue) staining (× 200) in the prefrontal cortex of adolescent rats; **(B)** Iba-1/DAPI (red/blue) staining (× 200) in the hippocampus of adolescent rats; **(C)** Iba-1/DAPI (red/blue) staining (× 200) in the prefrontal cortex of adult rats; **(D)** Iba-1/DAPI (red/blue) staining (× 200) in the hippocampus of adult rats; **(E)** Iba-1^+^ cell counts in the prefrontal cortex; **(F)** Iba-1^+^ cell counts in the hippocampus. The results are expressed as the mean ± SEM (*n* = 5 each group). ^#^ and ^###^ indicate *p* < 0.05 and *p* < 0.001 vs. C group, respectively; ^**^ and ^***^ indicate *p* < 0.01 and *p* < 0.001 vs. S group, respectively. AdoC, adolescent control group; AdoS, adolescent CUMS group; AdoS+M, adolescent CUMS and minocycline group; AduC, adult control group; AduS, adult CUMS group; AduS+M, adult CUMS and minocycline group.

Figure 4A shows the Iba-1 (as the microglia marker) expression in the prefrontal cortex in adolescent groups. Figure 4C shows the Iba-1 expression in the prefrontal cortex in adult groups. Fewer branches and larger cell bodies of the microglia in the prefrontal cortex were observed in the stressed group when compared with the C group and the S+M group in both adolescence and adulthood. Figure 4E shows the count of cells that positively express Iba-1 in the prefrontal cortex. We observed that the Iba-1-labeled microglial cells had a larger soma size and were more abundant in the AdoS group [*F*_(2, 12)_ = 31.58, *p* < 0.001] (*p* < 0.001) and the AduS group [*F*_(2, 12)_ = 9.64, *p* = 0.003] (*p* = 0.037), when compared with the AdoC group and the AduC group, respectively. Meanwhile minocycline treatment reversed the changes in the rats of the AdoS+M group (*p* < 0.001) and the AduS+M group (*p* = 0.003).

Figure 4B shows the Iba-1 expression in the hippocampus in the adolescent groups. Figure 4D shows the Iba-1 expression in the hippocampus in the adult groups. Fewer branches and larger cell bodies of the microglia in the hippocampus were observed in the stressed group when compared with the C group and the S+M group in both adolescence and adulthood. Figure 4F shows the count of cells that positively express Iba-1 in the hippocampus. In adolescence, the AdoS group [*F*_(2, 12)_ = 61.32, *p* < 0.001] had a significantly increased number of microglia when compared with the AdoC group (*p* < 0.001) and the AdoS+M (*p* < 0.001) group. The adult groups had the same change tendencies in that stress increased the number of microglia in the AduS group [*F*_(2, 12)_ = 10.02, *p* = 0.003] when compared with the AduC group (*p* = 0.035), and minocycline suppressed the activation in the AduS+M group (*p* = 0.002).

### Comparison of iNOS expression in the prefrontal cortex and hippocampus between groups

**Figure 5:**
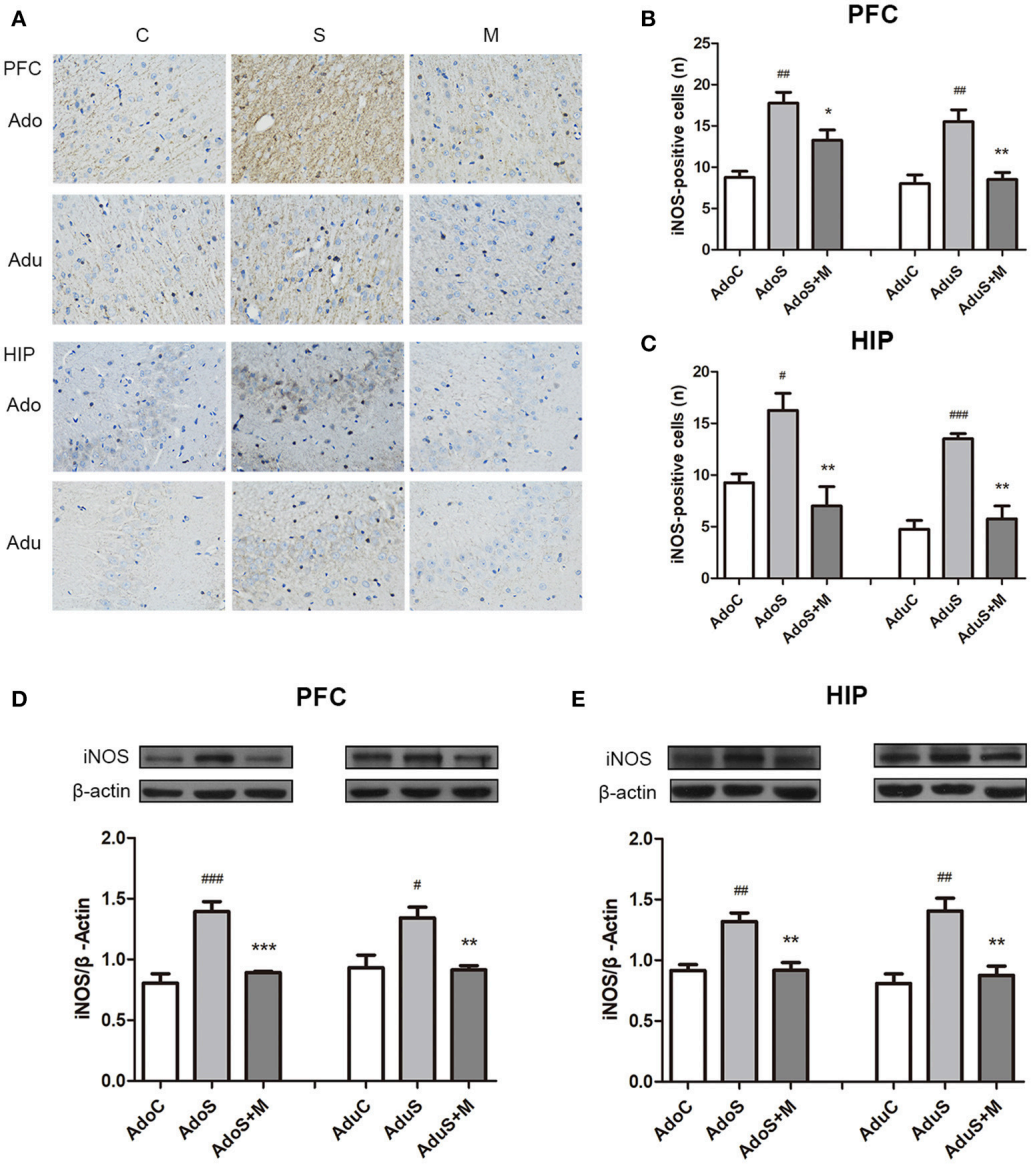
CUMS induced M1 marker iNOS expression in the prefrontal cortex and hippocampus of both adolescent and adult rats, while minocycline treatment reduced the expression. **(A)** iNOS expression in the prefrontal cortex and hippocampus in immunohistochemistry (× 400); **(B)** iNOS-positive cell counts in the prefrontal cortex; **(C)** iNOS-positive cell counts in the hippocampus; **(D)** Western blot analysis of iNOS in the prefrontal cortex; **(E)** Western blot analysis of iNOS in the hippocampus. Results are expressed as the mean ± SEM (*n* = 4 each group; *n* = 5 each group). ^#^, ^##^, and ^###^ indicate *p* < 0.05, *p* < 0.01, and *p* < 0.001 vs. C group, respectively; ^*^, ^**^, and ^***^ indicate *p* < 0.05, *p* < 0.01, and *p* < 0.001 vs. S group, respectively. AdoC, adolescent control group; AdoS, adolescent CUMS group; AdoS+M, adolescent CUMS and minocycline group; AduC, adult control group; AduS, adult CUMS group; AduS+M, adult CUMS and minocycline group.

Figure 5A shows the iNOS (M1 activation marker) expression in the prefrontal cortex and hippocampus in immunohistochemistry. Figure 5B shows the count of cells that positively expressed iNOS in the prefrontal cortex. In adolescent rats, CUMS could induce iNOS expression [*F*_(2, 9)_ = 15.76, *p* = 0.001] (*p* = 0.001), while minocycline treatment reversed the alteration (*p* = 0.049). In adult rats, the stress-exposed rats also had higher iNOS expression than the AduC group [*F*_(2, 9)_ = 13.12, *p* = 0.002] (*p* = 0.003). Minocycline treatment also reversed the alteration (*p* = 0.005). Figure 5C shows the count of cells that positively express iNOS in the hippocampus. In the adolescent groups, the AdoS group had higher iNOS expression than the AdoC group [*F*_(2, 9)_ = 10.03, *p* = 0.005] (*p* = 0.025) and the AdoS+M group (*p* = 0.005). In the adult groups, the expression of iNOS also increased in the AduS group [*F*_(2, 9)_ = 27.07, *p* < 0.001] (*p* < 0.001) when compared with the AduC group and the AduS+M group (*p* = 0.001).

Figure 5D shows the expression of iNOS in the prefrontal cortex in Western blot. In the adolescent groups, the AdoS group [*F*_(2, 12)_ = 24.15, *p* < 0.001] had higher levels of iNOS expression than the AdoC group (*p* < 0.001) and the AdoS+M group (*p* < 0.001). In the adult groups, the expression of iNOS increased in the AduS group [*F*_(2, 12)_ = 8.84, *p* = 0.004)] when compared with that in the AduC group (*p* = 0.01) and the AduS+M group (*p* = 0.008).

Figure 5E shows the iNOS expression in the hippocampus in Western blot. Increased iNOS expression was seen in the AdoS group [*F*_(2, 12)_ = 14.42, *p* = 0.001] when compared with the AdoC group (*p* = 0.001), but minocycline treatment normalized the alteration in the AdoS+M group (*p* = 0.002). The AduS group [*F*_(2, 12)_ = 13.99, *p* = 0.001] had higher levels of iNOS expression than the AduC group (*p* = 0.001). Similarly, minocycline reduced the expression of iNOS in the AduS+M group (*p* = 0.003) when compared with the AduS group.

### Comparison of expression of Jmjd3 and H3K27me3 between groups

**Figure 6:**
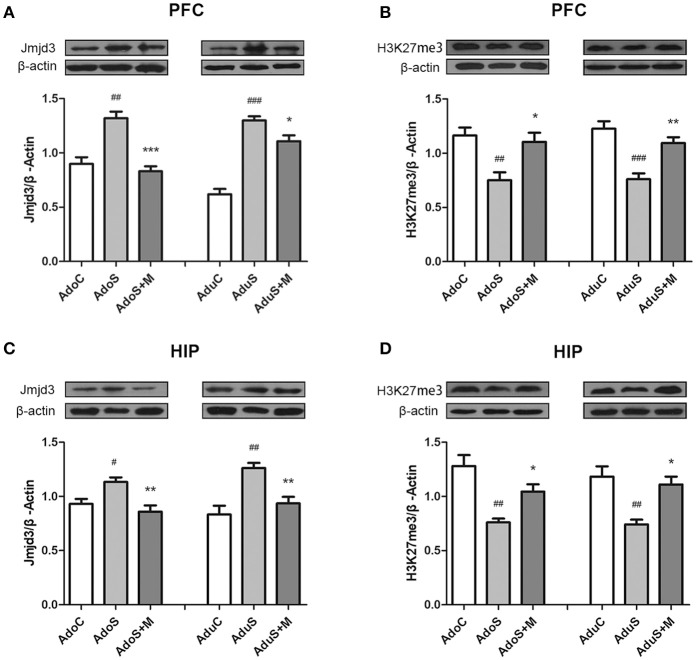
The expression of Jmjd3 and H3K27me3 in the prefrontal cortex and hippocampus detected by Western blotting. **(A)** Western blot analysis of Jmjd3 in the prefrontal cortex; **(B)** Western blot analysis of H3K27me3 in the prefrontal cortex; **(C)** Western blot analysis of Jmjd3 in the hippocampus; **(D)** Western blot analysis of H3K27me3 in the hippocampus. Results are expressed as the mean ± SEM (*n* = 5 each group). ^#^, ^##^, and ^###^ indicate *p* < 0.05, *p* < 0.01, and *p* < 0.001 vs. C group, respectively; ^*^, ^**^, and ^***^ indicate *p* < 0.05, *p* < 0.01, and *p* < 0.001 vs. S group, respectively. AdoC, adolescent control group; AdoS, adolescent CUMS group; AdoS+M, adolescent CUMS and minocycline group; AduC, adult control group; AduS, adult CUMS group; AduS+M, adult CUMS and minocycline group.

Figure 6A shows Jmjd3 expression in the prefrontal cortex in every group in adolescence and adulthood using Western blot. In the adolescent group, Jmjd3 levels showed significant group effects [*F*_(2, 12)_ = 21.23, *p* < 0.001]. Stress induced the expression of Jmjd3 in rats of the AdoS group (*p* = 0.001) more strongly than in the AdoC group. Nevertheless, minocycline decreased the level of Jmjd3 expression in the AdoS+M group when compared with the AdoS group (*p* < 0.001). In adult groups, increased Jmjd3 expression was seen in the AduS group [*F*_(2, 12)_ = 54.72, *p* < 0.001] (*p* < 0.001) when compared with the AduC group. Minocycline reversed the alteration (*p* = 0.037 AduS group vs. AduS+M group).

Figure 6B shows H3K27me3 expression in the prefrontal cortex in both adolescent and adult groups. The AdoS group had a markedly decreased H3K27me3 level when compared with the AdoC group [*F*_(2, 12)_ = 8.56, *p* = 0.005] (*p* = 0.006). Whereas, animals in the AdoS+M group showed a significantly higher H3K27me3 level when compared with the AdoS group (*p* = 0.017). In adult groups, stress induced the reduced expression of H3K27me3 in the AduS group [*F*_(2, 12)_ = 16.38, *p* < 0.001] (*p* < 0.001) when compared with the AduC group, while minocycline treatment could reverse the decreased expression in the AduS+M group (*p* = 0.005).

Figure 6C shows Jmjd3 expression in the hippocampus of adolescent and adult groups using Western blot. The same change tendencies in Jmjd3 expression in the hippocampus were observed as in the prefrontal cortex. Stress induced the over-expression of Jmjd3 in rats from the AdoS group [*F*_(2, 12)_ = 8.24, *p* = 0.006] (*p* = 0.033) when compared with the AdoC group. However, minocycline treatment decreased the level of Jmjd3 expression in the AdoS+M group when compared with the AdoS group (*p* = 0.005). In the adult groups, the AduS group [*F*_(2, 12)_ = 12.24, *p* = 0.001] (*p* = 0.001) had an increased Jmjd3 expression when compared with the AduC group; however, minocycline reversed the alteration (*p* = 0.009).

Figure 6D shows H3K27me3 expression in the hippocampus in both adolescent and adult groups, which had the same change tendency as the prefrontal cortex. The AdoS group had a markedly decreased H3K27me3 level when compared with the AdoC group [*F*_(2, 12)_ = 12.70, *p* = 0.001] (*p* = 0.001). Nevertheless, animals in the AdoS+M group showed a significant increase in H3K27me3 levels when compared with the AdoS group (*p* = 0.044). Similarly, the AduS group had lower H3K27me3 levels than the AduC group [*F*_(2, 12)_ = 10.35, *p* = 0.002] (*p* = 0.003). Meanwhile, minocycline treatment reversed the decrease in the AduS+M group when compared with the AduS group (*p* = 0.01).

## Discussion

The present study revealed that CUMS in the adolescent period induced short-term and persistent depressive-like behaviors, high levels of pro-inflammatory cytokines, microglial activation, and increased Jmjd3 and decreased H3K27me3 expression in the prefrontal cortex and hippocampus. The results indicate that the alteration of Jmjd3 and H3K27me3 expression plays a critical role in susceptibility to depressive-like behaviors in rats that had early-life stress experiences.

Previous studies suggested that social-psychological stress exposure in early-life increases the risk of mood disorders (38). Our previous study revealed that CUMS and acute stress in early life induce long-term dysfunctional behaviors, which supports the conclusion that early-life stress has long-lasting effects on individual behaviors (33, 39). Consistent with the above study, CUMS in the adolescent period induced depressive-like behaviors and memory damage in both adolescent and adult animals in our present study. Moreover, minocycline, the microglial activation inhibitor, attenuated the abnormalities of behaviors in adolescent and adult groups, which indicates that inhibition of microglial activation could relieve the depressive-like behaviors. These results suggest that early-life stress gives rise to long-lasting behavioral disorders (40, 41) and also that microglial activation is involved in behavioral abnormalities (42).

An increasing number of studies suggest that some pro-inflammatory cytokines are closely linked to the pathogenesis of depression under stress by a mechanism in which cytokines decrease the levels of serotonin, noradrenaline, and dopamine in the limbic system and stimulate the hypothalamic-pituitary-adrenal (HPA) axis to release glucocorticoids (2). In addition, pro-inflammatory cytokines could induce sickness-like behaviors independently; for example, IL-1β decreased locomotor activity (35). Cytokines also initiate the cycle of pro-inflammatory responses by giving rise to a cascade of inflammatory cytokine responses when the patient is suffering from psychological stress (43). The present study showed that CUMS induced significantly increased levels of IL-6 and IL-1β in both the hippocampus and prefrontal cortex. However, minocycline treatment attenuated these alterations. More importantly, adult rats in the CUMS group showed higher levels of IL-6 and IL-1β in both the hippocampus and prefrontal cortex along with depressive-like behaviors. Hence, our results demonstrate that higher levels of pro-inflammatory cytokines in the brain induced by CUMS during the adolescent period are associated with vulnerability to depression in adulthood.

Previous studies pointed out that classical microglial activation facilitates the occurrence of depression by pro-inflammatory cytokines during which stress plays an important role. First, stress induces HPA axis activation, and massive amounts of glucocorticoids are released into the prefrontal cortex and hippocampus, where they are susceptive to the corticosterone surge. Microglia express a large number of glucocorticoid receptors, whereupon stress induces microglial activation. Secondly, stress induces pro-inflammatory cytokine (IL-1β) over-expression in the CNS leading to the activation of microglia (44, 45). In order to clarify the role of microglial activation in neuroinflammation from adolescence to adulthood caused by CUMS in adolescence, the present study examined the Iba-1 and iNOS expression in the prefrontal cortex and hippocampus in both adolescent and adult rats. The results show that Iba-1 and iNOS are over-expressed in the prefrontal cortex and hippocampus of stressed rats when compared with their expression in unstressed rats. Furthermore, the morphology of microglia underwent a noticeable change from a ramified morphology to an amoeboid shape in stressed rats. These results are consistent with previous studies that showed that microglia increased in number, and hyper-ramified properties arose in response to chronic stress (46). Moreover, similar results were detected in adult rats, which support the argument that early-life stress could lead to long-term pro-inflammatory effects and classical microglial activation (47). Our results support the “two-hit” theory of the involvement of neuroinflammation in the etiology of mental disorders, which proposes that early-life stress primes or sensitizes microglia. Stress in later life can provoke the sensitized microglia, which can generate an exaggerated response and increase the risk of development of a mental disorder (44). Therefore, our results expanded the conclusion that stress-induced microglial activation in early-life is closely linked to the susceptibility to depression. Moreover, the present study illustrates the role of minocycline in microglial activation in early-life stress-induced depression. Consistent with previous studies (31, 48), minocycline administration reduced depressive-like behaviors and inhibited the expression of pro-inflammatory cytokines. These results imply that minocycline has the potential to be used as a treatment for depression by targeting microglial activation.

Recent research has demonstrated that Jmjd3 can remove the tri-methylation and di-methylation marks of H3K27 and then lead to gene expression. Most importantly, Jmjd3 was induced both by inflammation and by stress (23, 49). Jmjd3 decreased the level of repressive H3K27me3 marks at the promoters of NF-κB-driven inflammatory genes such as IL-1β (50). Thus, the higher expression of Jmjd3 participated in inflammation by enhancing the transcription of inflammatory genes via the NF-κB signaling pathway (49). In addition, NF-κB transcription factors were the driving forces for microglial activation (50). Hence, Jmjd3 was a key factor in a cascade of inflammatory cytokine response (23, 28, 49, 50). In this study, we investigated the alterations of Jmjd3 and H3K27me3 expression under stress and found that CUMS induced increased Jmjd3 expression and decreased H3K27me3 expression in the prefrontal cortex and hippocampus. These results imply that the downregulation of H3K27me3 by Jmjd3 mediated the expression of cytokines. It is important to note that minocycline, as an inhibitor to microglial activation, could reverse all the alterations in both adolescent and adult animals. Our results, for the first time, highlighted that Jmjd3 plays a key role in the inflammatory response to CUMS and mediates the susceptibility to depression by regulating microglial activation and pro-inflammatory cytokine expression in the prefrontal cortex and hippocampus.

## Conclusions

Exposure to CUMS in the adolescent period induced long-term depressive-like behaviors, increased pro-inflammatory cytokine expression and microglial activation, increased Jmjd3 expression, and decreased H3K27me3 expression in the prefrontal cortex and hippocampus of adolescent and adult rats. Meanwhile, minocycline, a microglial activation inhibitor, mitigated all the alterations. Our study suggests that Jmjd3 might be involved in the susceptibility to depression by modulating the microglial activation and pro-inflammatory cytokine expression.

## Limitation

This study has limitations. First, primed microglia have two functional subtypes, “classical activation” and “alternative activation.” The present study only discussed the “classical activation,” and no indicators of the “alternative activation” were included. Second, Jmjd3 is an important epigenetic element in microglial activation. Lack of a Jmjd3 inhibitor limits the understanding about the role of Jmjd3 in susceptibility to depression induced by early-life stress.

## Ethics statement

In the handling and care of all animals, the international guiding principles for animal research, as stipulated by the World Health Organization (WHO) Chronicle (World Health Organization, 1985) and as adopted by the Laboratory Animal Center, Shandong University, were followed.

## Author contributions

FP conceived and designed the experiments. RW performed most of the experiment and analyzed the data. FP and RW wrote and refined the article. WW participated in the animal modeling and behavioral experiments. JX assisted in laboratory work and figure preparation. DL and HJ supervised the acquisition of results.

### Conflict of interest statement

The authors declare that the research was conducted in the absence of any commercial or financial relationships that could be construed as a potential conflict of interest.
