# Supplementary material for: Dynamic Effects of Early Adolescent Stress on Depressive-Like Behaviors and Expression of Cytokines and JMJD3 in the Prefrontal Cortex and Hippocampus of Rats
Source: Front Psychiatry. 2018 Oct 11;9:471. doi: 10.3389/fpsyt.2018.00471 (PMC6193509; doi:10.3389/fpsyt.2018.00471)
Supplement: Supplementary file 1 [file Table_1.DOCX]

**CUMS+Mino**

**Behavioral**

**Tests**

**CUMS**

**Modeling**

**Sacrifice**

**Acclimatization**

**90d**

**49d**

**Behavioral**

**Tests**

**84d**

**55d**

**21d**

**28d**

**Adolescent groups**

**Sacrifice**

**Adult groups**

The schematic diagram of animal modeling, drugs administration and behavioral tests
